# The Oneness of Dentistry

**Published:** 1860-10

**Authors:** 


### THE ONENESS OF DENTISTRY.

Operative and mechanical dentistry should be as strictly a unit, in scientific attainments, as the human body is unitary in the aggregation of its organs. As no work can be well performed without the justly balanced consent of head, heart and hand, so nothing perfect can emanate from a fragmentary Dentist.

I regret to see those who deem themselves at the head of the teachers' department in our profession, arraying hand against foot, and eye against ear, just as if all were not necessary to the completion of the good to the race with which we, as a profession, are charged. Let us not perpetuate the necessity to use us, on the part of patients, by a half expressed skill, by dividing and separating that which was designed to go hand in hand, and accomplish the nearest approach to original *soundness*, of parts and of whole bodies, the sure endowment of all, where no discordant influence is permitted to play its baleful part.

Do you ask how we shall arrive at this very desirable state of our professional body ?

I answer,—by being so in earnest to do our highest, *every time*, that we shall forget the besetment of even *thinking* of the " fee," till *after* the work is completed, in our highest and best manner. No doubt this will be "rule prohibitory" to some who acknowledge and call themselves dentists. But I do most emphatically state it, as the deliberate conclusion of my mind, after " due trial " and consideration. Most of the trials of my professional life crept in at the door of " determining, before hand, *just* what it was going to *cost*, to patient, in cash, and to me, in effort ;" and I am willing to stand or fall upon the " turn of this die."

No one is entitled to *assert*, where he can *demonstrate*, nor to tarry in the regions of " opinion " and " belief," when he can have the clear and satisfactory light of *experience*, constituting the sure basis of well defined knowledge, to illuminate and guide him.

A higher " *morale* " *must* be taken by our profession, as a whole, to keep it from degenerating to the merest " Tinkerdom."

What would my *beloved* brethren think of me, if I were to express, as the inmost conviction of my mind, that you could count, on your fingers, (and have a portion of the enumeration table left for future use) all the *legitimate* dentists on the continent ? Or, still further, that, as yet, we have not, in all our ranks, *one* whole-souled and perfect dental type, whom it would be safe to follow, in *all* his practices ?

If, then, we have had as yet no perfect work, what is the inference, in view of all the very excellent advances that have been made in the various departments of our specialty, to plainly so state and maintain ? Simply this, that the time has not come when there is an end to our usefulness, in an increasing degree, leaving ample field for advencement to an indefinite number of earnest, zealous workers, in one and all of the *departments* of our *now* recognized, necessary calling.

He who gets an early start in the morning, is better able to keep up with the progress of the day, than he who deems himself quite competent to the duties he has not tried, and, presuming upon this, delays his preparation, till the day is far advanced. So he who has made the most of his spare moments, and has played the sluggard, or " nice young man," the least, in the days of his preparation for professional life, will, assuredly, stand in advance of any who, having facilities, depended upon them for a proper fitness for valuable service, instead of acquainting himself with the principles which, inevitably, underlie all legitimate and remunerative practice. Please notice, I place the first requisite in its proper place, that is, fitness before fees; and woe be to him who has the audacity to reverse the order, hoping, thereby, to elevate either himself or his profession. It is like endeavoring to build a cone, with apex for base, and will as surely topple over. Labor earnestly, unremittingly; and just as sure as you faint not by the way, a success, exactly commensurate with the effort bestowed to fit you to deserve it, will be yours to enjoy.

All who stop short of a fair degree of fitness, before commencing the responsibilities of practice, do themselves and the profession an irreparable mischief. By that very honesty of heart by which they should be blessed, they are cursed bitterly; because, their standard being low, they think they have attained all the attainable excellence, and are " as good as their neighbors,"—all of which being true, does not prove them honorable members of an honorable calling. All this, " I guess it will do," and " we'll make it go " sort of work, is but a plain enunciation that neither work nor operative is fit for use at all. And the sooner such practices and practicers are completely severed from all connection with the profession, the sooner will the " weights " be " laid aside," and true progress come to the most progressive of sciences.

Rule, to determine who is fit, or *becoming* fit, for the high responsibilities of a free exercise of the function of Dentist:

Make every step of your service, in study and practice, a matter of highest duty—from inception to entire fulfillment, according to your *own* sense of perfectibility, without one lapse being left unretrieved.

All who do so comport themselves, will soon stand in the front ranks, for usefulness and respectability; and will have laid, surely and peacefully, the foundation of a higher happiness to themselves and those they serve. Thus they may *feel* that they are fulfilling the "dispensation committed to them." And I would reject, as interlopers, thieves and robbers, all who have no "mission" to *feel* and acknowledge.

